# A New Sampling Approach for the Detection of Swine Influenza a Virus on European Sow Farms

**DOI:** 10.3390/vetsci9070338

**Published:** 2022-07-04

**Authors:** Kathrin Lillie-Jaschniski, Marina Lisgara, Emanuela Pileri, Agnes Jardin, Eduardo Velazquez, Monika Köchling, Michael Albin, Carlos Casanovas, Vassilis Skampardonis, Julia Stadler

**Affiliations:** 1 Ceva Tiergesundheit, Kanzlerstraße 4, 40472 Düsseldorf, Germany; monika.koechling@ceva.com; 2 Ceva Hellas LLC, 16341 Athens, Greece; marina.lisgara@ceva.com; 3 Ceva Salute Animale, 20864 Agrate Brianza, Italy; emanuela.pileri@ceva.com; 4 Ceva Santé Animale, 10 Avenue de la Ballastière, 33501 Libourne, France; agnes.jardin@ceva.com; 5 Ceva Animal Health Ltd., Amersham HP7 9FB, UK; eduardo.velazquez@ceva.com; 6 Ceva Animal Health Ltd., Ladegaardsvej 2, 7100 Vejle, Denmark; michael.albin@ceva.com; 7 Ceva Salud Animal, 08028 Barcelona, Spain; carlos.casanovas@ceva.com; 8 Department of Epidemiology, Biostatistics and Economics of Animal Production, School of Veterinary Medicine, University of Thessaly, 43132 Karditsa, Greece; bskamp@uth.gr; 9 Clinic for Swine, Centre for Clinical Veterinary Medicine, Faculty of Veterinary Medicine, Ludwig Maximilian University, 75000 Munich, Germany; j.stadler@med.vetmed.uni-muenchen.de

**Keywords:** swine influenza, influenza A virus, nasal swabs, sampling, diagnosis

## Abstract

**Simple Summary:**

Due to concerns in public health and its negative impact on the pig industry the need for Influenza A virus (IAV) surveillance is rising. The gold standard procedure for detecting IAV is to sample acutely diseased pigs. Endemic infections with unspecific clinical signs and low disease prevalence need new approaches. Our study aimed to evaluate a standardized sampling procedure for the detection of IAV in epidemically and endemically infected farms. We performed a cross-sectional study in 131 farms investigating three different age groups per farm in 12 European countries. The results of our investigation indicate that 10 nasal swabs each in suckling piglets, weaners and middle of nursery is a valuable tool for influenza detection and identification of subtypes. However, for farms with a lower prevalence than 15% it is advisable to either increase the number of nasal swabs in each age group or to use group sampling methods. Interestingly, different subtypes were found in different age groups. Thus, our study underlines that sampling of different age groups is mandatory to obtain a comprehensive overview on all circulating variants on farm. In addition, our results highlight that sampling strategies should also consider piglets without obvious clinical signs for IAV infection.

**Abstract:**

Swine influenza A virus (swIAV), which plays a major role in the porcine respiratory disease complex (PRDC), is eliminated from the respiratory tract within 7–9 days after infection. Therefore, diagnosis is complicated in endemically infected swine herds presenting no obvious clinical signs. This study aimed to investigate the right time point for sampling to detect swIAV. A cross-sectional study was performed in 131 farms from 12 European countries. The sampling protocol included suckling piglets, weaners, and nursery pigs. In each age group, 10 nasal swabs were collected and further examined in pools of 5 for swIAV by Matrix rRT-PCR, followed by a multiplex RT-PCR to determine the influenza subtype. SwIAV was detected in 284 (37.9%) of the samples and on 103 (78.6%) farms. Despite the highest number of animals with clinical signs being found in the nursery, the weaners were significantly more often virus-positive compared to nursery pigs (*p* = 0.048). Overall, the swIAV detection rate did not significantly differ between diseased or non-diseased suckling and nursery piglets, respectively; however, diseased weaners had significantly more positive pools than the non-diseased animals. Interestingly, in 9 farms, different subtypes were detected in different age groups. Our findings indicate that to detect all circulating swIAV subtypes on a farm, different age groups should be sampled. Additionally, the sampling strategy should also aim to include non-diseased animals, especially in the suckling period.

## 1. Introduction

Influenza A infections in swine (swIAV), as part of the porcine respiratory disease complex (PRDC), can cause severe clinical signs that lead to decreased performance in growers and fatteners [1] and can also have a negative impact on the reproductive performance of the sow herd [2]. As pigs also harbor receptors for the human and avian influenza virus, they play a role as a “mixing-vessel” for new reassortants, harboring the danger of zoonotic potential [3,4,5]. The use of vaccines often forms part of influenza prevention in swine herds. To enable the selection of the correct vaccination strategy, an exact knowledge of the subtypes involved, not only on the individual farms but also within the region, is necessary [6,7]. Today, the main two strategies to detect influenza on farms include individual samples from diseased animals, which is still viewed as the “gold standard” [8], or cross-sectional group samplings. In individual samplings, influenza detection is mainly performed by analyzing the lungs during necropsy, bronchoalveolar lavage, and, very often, nasal swabs are taken from acutely diseased animals exhibiting sneezing, coughing, and/or high fever symptoms. Nasal swabs can either be examined as single swabs or are often in pools of five for further investigation. This reduces the cost of the PCR without significant loss of sensitivity. Commonly used group-sampling methods include oral fluids, while the recent publication by Garrido-Mantilla et al. in 2019 [9] resulted in the use of udder wipes to detect the swIAV virus on farms. Previously, group samplings were mainly used for monitoring purposes but, as influenza very frequently persists endemically on farms while not always leading to obvious clinical signs, the need for convenient but reliable sampling methods has increased. Particularly in endemically infected herds, the right choice of animals to sample is crucial, as the recurrent circulation of the influenza strain means that some of the sow population will already have had contact with the virus. These previously infected sows can transfer maternally derived antibodies (MDA) to their piglets [10]. MDA can prevent clinical signs in suckling piglets that either get infected by virus-shedding sows, by airborne transmission, by farm workers [11], or from other sources. However, clinically protected suckling piglets will shed the same amount of virus particles after infection as do piglets that did not receive MDA [12,13]; therefore, these clinically unsuspicious suckling piglets can transfer the virus into the nursery. It has been shown that after introduction into the nursery, depending on the strain involved, the group sizes, and the amount of mixing among animals, the infection dynamics can vary greatly [14]. Although these infected piglets do not show obvious clinical signs, coinfections with bacteria such as *Streptococcus suis* [15] or *Glaesserella parasuis* [16], or with other viral agents such as porcine reproductive and respiratory syndrome virus (PRRSV) [17], can lead to severe clinical signs [18]. Consequently, these signs appear later in the nursery after the primary infection with swIAV, which has often been eliminated by the animals at the time point of visible clinical signs. As the virus can only be detected up to 7–9 days after infection [19], samples taken from these acutely diseased animals can appear negative in the performed influenza A PCR. Another factor that can complicate the diagnosis is the co-circulation of multiple strains on farms with different prevalence and infection dynamics [20].

The aim of the present study was to establish an efficient sampling approach for piglets on farms, either when an acute influenza outbreak is suspected or in an endemic course of the disease. To gain an overall overview of the infection dynamics, as well as the subtypes circulating on farms, a cross-sectional study investigating three different age groups was performed in 12 different European countries.

## 2. Materials and Methods

### 2.1. Farm Selection and Sampling Protocol

**Figure 1:**
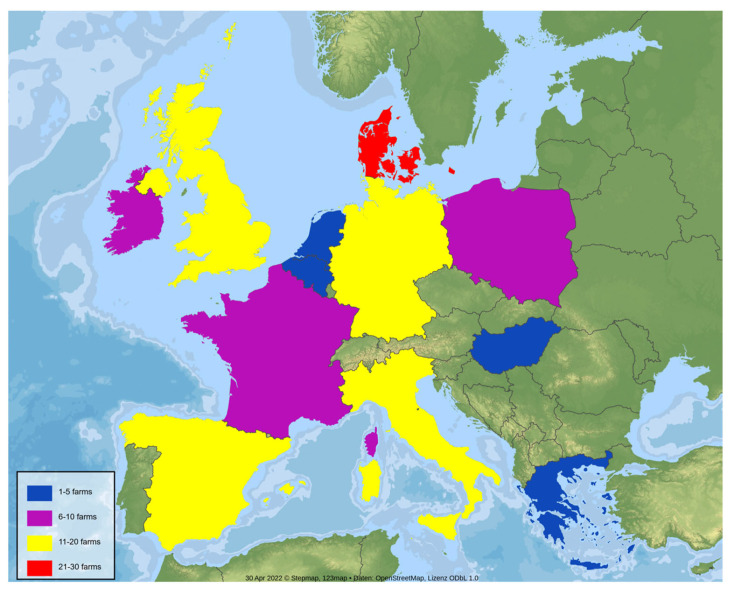
The 12 European countries that participated in the study, colored and sized differently according to the number of farms that were sampled in each country (blue: 1–5 farms, purple: 6–10 farms, yellow: 11–20 farms, red: 21–30 farms). This map was constructed using StepMap^®^.

A cross-sectional study was conducted in 12 different European countries. The farms were selected using the following inclusion criteria: farms with either acute clinical signs suggesting swIAV, including dyspnea, coughing, sneezing, nasal discharge, anorexia, and/or lethargy (epidemically infected farms), or farms suspecting endemic swIAV circulation, due to either reproductive failure and fever in sows or recurrent respiratory distress in the nursery (endemically infected farms). Farm owners voluntarily participated in the investigation. Between January and December 2021, a total of 131 farms were examined once (Figure 1).

The sampling protocol consisted of 10 nasal swabs in each of the following age groups: suckling piglets (1–4 weeks old), piglets around weaning (4–6 weeks old) and animals in the middle/end of the nursery period (7–9 weeks old). In the suckling period, one strong piglet per litter out of 10 litters was sampled. In piglets after weaning and in the middle/end of the nursery period, one piglet per pen and around 10 pens were sampled. The sample size was calculated using a fixed design prevalence of 15%, for a minimum of 95% probability of detection, assuming a diagnostic sensitivity and specificity of 90% and 100%, respectively. Therefore, the target number of sampled animals per farm was 30 animals within three age groups [9]. To maximize the probability of IAV detection, whenever possible, those animals showing clinical signs suggestive of IAV infection were preferentially sampled.

The collection of nasal swabs was performed by inserting the swab 2–4 cm into both nostrils of each piglet and rotating it 360 degrees. Swabs were placed into a plastic tube with 2 mL of viral media (Virocult^®^). Each tube contained a pool of 5 individual swabs, resulting in 6 pooled samples per farm. Plastic tubes were sent under cooling conditions (using ice packs) to the laboratory for RNA extraction and testing. In the laboratory, each pool of nasal swabs in the first step was investigated by real-time PCR (rRT-PCR) targeting the matrix gene of influenza A. Results with a cycle threshold (Ct) value < 38 were considered positive, and a Ct > 38 was considered negative. All positive samples with a Ct value below 30 were further investigated via multiplex RT-PCR to determine the influenza A subtype by hemagglutinin (HA) and neuraminidase (NA) gene amplification, targeting H1av, H1pdm, H1hu, H3hu, N1 (including N1all and N1pdm), and N2. If only the HA could be determined, samples were counted as typable. IAV rRT-PCR and multiplex RT-PCR were performed according to the protocol of Henritzi et al. 2016 [21].

The preliminary reported influenza status of the herds (endemically and epidemically), the farm size, which was categorized into three groups (50–499 sows, 500–999 sows, and 1000–10 000 sows) [22], influenza vaccination status, and the clinical signs shown by the sampled animals were recorded (no clinical signs or clinical signs). Herd examination also included a technical questionnaire, assessing the production and management parameters and details regarding the sampled age groups.

### 2.2. Statistical Analysis

All statistical analyses were performed using Stata 13.1 (Stata Statistical Software, College Station, TX, USA). The significance level was set at 0.05.

Descriptive statistics of the collected data were produced. Chi-square tests were performed in order to investigate whether herd-level positivity differs according to the influenza status of the herd, vaccination status of the herd, and farm size. The investigation of the existence of an association between the rRT-PCR results and the collected information on potential risk factors was performed with the use of a three-level mixed-effect logistic regression model. The rRT-PCR result (either positive or negative) was the dependent variable, while the information/parameters collected were the independent variables and were offered as explanatory variables in the model. Recorded information included the age category of the piglets in the sampled pools, the influenza status of the herds (endemic, epidemic), the farm size, influenza vaccination status, and clinical signs of the sampled animals (no clinical signs or clinical signs). Our data were organized in a hierarchical structure; sampled piglets were clustered in farms and farms were clustered in countries; thus, random effect terms were incorporated to account for the within-herd and within-country correlation of observations resulting from the multilevel design of the study. For the screening process of candidate variables for multivariable modeling, a bi-variable approach was used, as suggested by Martin (1997) [23]. The age group of the sampled piglets was hypothesized to be important for the final model; thus, it was forced in all models during the screening process [14,24]. All independent variables were initially screened one by one in bivariate mixed-effects logistic regression models, along with the piglets’ “age group”. During this screening process, the level of significance was set at 0.25 [25]. Subsequently, variables with *p* < 0.25 were offered to a full regression model at the same time, which was further reduced by backward elimination [26] until only significant variables at the 5% level remained. Pairwise interactions between the remaining variables were created and were offered one at a time to the model. Lastly, previously excluded variables were offered one by one to the final model, to avoid omitting any variable that could add significantly to the model.

Subsequently, the above model was adapted accordingly and rerun thrice, within each piglet age group category, to investigate potential differences in the probability of occurrence of a positive rRT-PCR result between samples with and without apparent clinical signs, within samples from suckling piglets, weaners, and nursery piglets, respectively.

Additionally, in order to investigate the existence of a potential difference in rRT-PCR-positive results with a cycle threshold (Ct) value below 30, between the different sampled age groups of piglets, a mixed-effects logistic regression model was employed. The dependent variable represented the positive rRT-PCR results with either a Ct value > 30 and <38 or <30. The procedure for model-building and the selection of explanatory independent variables followed that described above.

Finally, as sampling was targeted primarily in piglets presenting clinical signs indicative of swine influenza, we were interested in investigating the degree of difficulty in finding these symptoms in the sampled age-group categories. In other words, we investigated potential differences in the probability of detection of the clinical signs between the sampled age categories of piglets. For this purpose, a three-level mixed-effect logistic regression model was employed, with the presence or absence of any clinical signs in the pool of sampled piglets as the dependent variable and the age-group category as the independent variable, while random effects terms at country and farm level were incorporated as well.

## 3. Results

A total of 131 farms fulfilled the inclusion criteria and could be incorporated into the present study. Overall, 750 pools of nasal swabs were analyzed for IAV.

### 3.1. Clinical Signs

**Figure 2:**
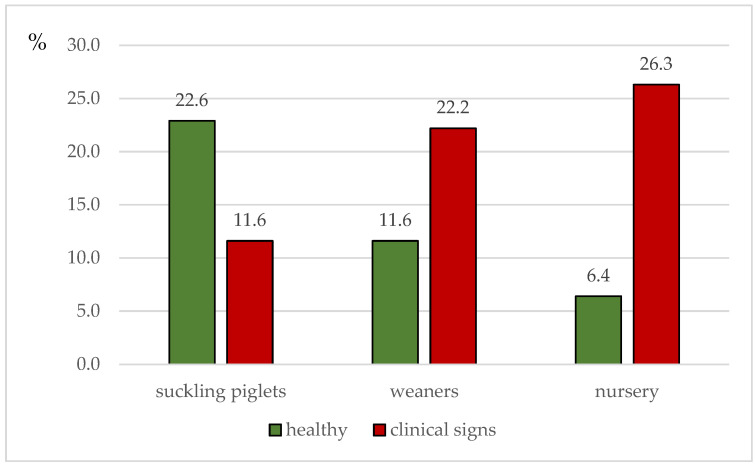
The percentage of piglets with and without clinical signs indicative of swine influenza (including, dyspnea, coughing, sneezing, nasal discharge, anorexia, and/or lethargy) among suckling piglets, weaners, and nursery pigs in relation to all samples (*n* = 750).

In the suckling period, the percentage of animals showing clinical signs indicative of IAV infection was 33.6% (87/259), whereas, in weaners, 64.7% (159/246) of the pooled samples originated from diseased animals. In the nursery, clinical signs were obvious in 80.4% (197/245) of the sampled animals (Figure 2).

Following the initial screening process, four variables, namely, the “age group category”, “herd size category”, “course of disease (acute/endemic)” and “clinical signs in the sampled pools” variables were eligible for and included in the full model. After the model-building processes, only the “age group category” and “clinical signs in the sampled pools” retained significance and remained in the final model. Their interaction was non-significant (*p* = 0.771).

Regarding the potential differences between the age group categories in terms of the probability of finding and sampling piglets with clinical signs suggestive of swine influenza, the three-level mixed-effect logistic regression model resulted in the following associations: testers were 8.18 times (*p* < 0.001, 95% C.I.: 4.80; 13.94) more likely to find animals with clinical signs in the weaners compared to the suckling piglets, 28.37 times (*p* < 0.001, 95% C.I.: 15.09; 53.33) more likely to find piglets with clinical signs in the nursery compared to the suckling period, and 3.46 times (*p* < 0.001, 95% C.I.: 2.05; 5.87) more likely to detect animals with clinical signs in the nursery compared to weaners (Figure 2).

### 3.2. IAV rRT-PCR Results at Farm Level

IAV was found by rRT-PCR in 103 (78.6%) out of the 131 investigated farms, in at least one of the samples (Table 1). Regarding the farm characteristics, 46 (82.1%) of the 59 farrow-to-finish farms and 58 (80.6%) of the 72 farrowing farms with attached nursery units were IAV-positive. In total, 41 epidemic farms and 90 endemic farms were included in this survey. Out of the epidemic farms, 82.9% (*n* = 34) and 76.7% (*n* = 69) of the endemic farms tested positive for Influenza A. The median herd size over all herds was 700 sows. Overall, 74.5% (*n* = 35) of the 47 farms ranging between 50 and 499 sows were positive for IAV, 68.2% (*n* = 30) of the 44 farms of between 500 and 999 sows, and 92.5% (*n* = 37) of the 40 farms between 1000 and 9000 sows. Sow vaccination (either reproductive or in a mass vaccination protocol) was implemented in 71 farms, of which 83.1% were IAV-positive, whereas of the 60 non-vaccinated herds, 80% were positive for IAV.

There was no statistically significant association between influenza A positivity at the herd level and the disease status of a farm (epidemic or endemic) (*p* = 0.913), the vaccination status (vaccinated/non-vaccinated sows) (*p* = 0.747), or the farm size (*p* = 0.485).

### 3.3. IAV rRT-PCR Results on Sample Level

Of the 750 investigated samples, 284 (37.9%) were positive for IAV. The percentage positivity for IAV for the suckling period, weaners, and nursery were 32.8 (85/259), 43.1% (106/246), and 38% (93/245), respectively. Pooled samples from weaners of 5–6 weeks of age (w.o.a.) showed no difference (*p* = 0.45) in terms of the odds of a positive PCR result, compared to suckling piglets (1–4 w.o.a.), and pooled samples from nursery pigs (7–9 w.o.a.) showed no difference (*p* = 0.28) in terms of the odds of a positive PCR result compared to suckling piglets. However, samples from weaners were 1.56 times more likely to be positive (*p* = 0.048, 95% CI: 1.004401; 2.42) compared to samples from nursery pigs (Table 2).

There was no statistically significant association between the influenza A positivity of samples and the disease status of a farm (epidemic or endemic) (*p* = 0.248), the vaccination status (vaccinated/non-vaccinated sows) (*p* = 0.72), and the farm size (*p* = 0.07).

**Figure 3:**
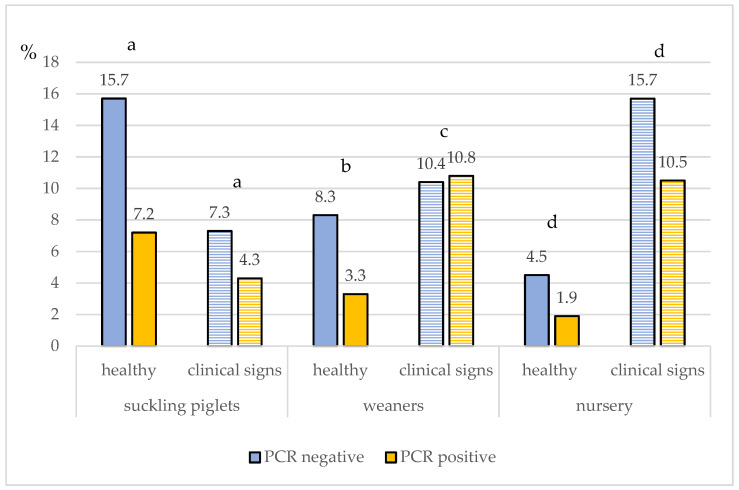
The percentage of positive and negative pools in influenza A-PCR among suckling piglets, weaners, and nurseries with and without clinical signs. Different superscripts within each age group indicate a significant difference (*p* < 0.05) between samples with “healthy” animals and animals with “clinical signs”.

Pooled samples from piglets with clinical signs were 3 times (*p* < 0.001, 95% CI: 1.82; 4.94) more likely to be positive, compared to pooled samples from piglets without clinical signs. The results of the mixed-effects logistic regression models within each age group category detected no significant difference in the odds of a positive RT-PCR result, neither between pooled samples from healthy and clinically diseased suckling piglets (*p* = 0.195, OR = 2.74, 95% CI: 0.59; 12.62) nor between pooled samples from healthy and clinically diseased piglets from the nursery (*p* = 0.103, OR= 4.63, 95% CI: 0.73; 29.25). However, a statistically significant difference in the odds of a positive RT-PCR result between pooled samples from healthy and clinically diseased weaners (*p* = 0.031) was detected. Particularly, samples originating from weaners with clinical signs were approximately 7 (OR = 7.12, 95% CI: 1.2; 42.26) times more likely to be positive, compared to weaners without clinical signs (Figure 3).

In terms of the Ct values of the samples, 63.5% (54/86) of the positive samples from suckling piglets showed a Ct value below 30, allowing us to perform a subtyping multiplex RT-PCR. Of the weaners, 60.4% (64/106), and in the nursery, 50.5% (47/93) of the positive samples showed Ct values below 30.

In the mixed-effect logistic regression model for the investigation of the association of the probability of positive rRT-PCR results with either a Ct value > 30 and <38 or <30, with potential risk factors among the recorded parameters, only age group and clinical signs in the sampled pools were eligible for inclusion in the full model. However, none of these variables retained significance at the 0.05 level in the final model. Thus, there was no difference in the odds of occurrence of a Ct value > 30 and <38 or <30 among positive samples, both between any age group category (*p* = 0.051, *p* = 0.069, and *p* = 0.969), in suckling piglets vs. weaners, in suckling piglets vs. nursery, and in nursery vs. weaners, respectively, and between pooled samples with or without clinical signs (*p* = 0.183).

### 3.4. IAV Subtyping Results at Farm Level

**Figure 4:**
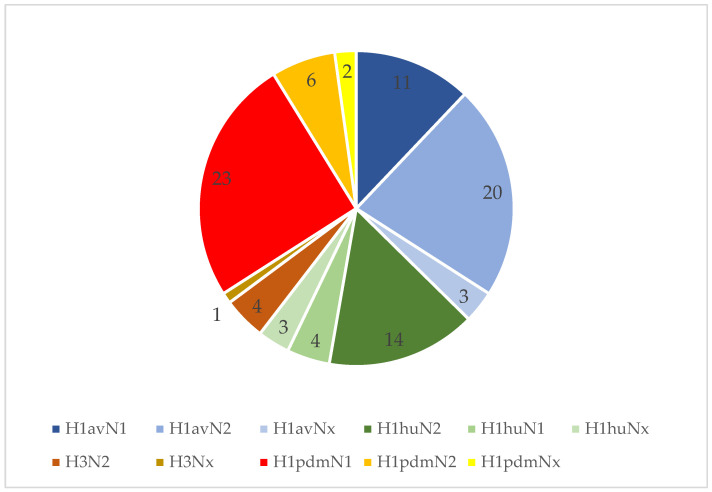
Subtypes detected on 78 of the 131 tested farms.

In 78 (75.7%) of the 103 IAV rRT-PCR-positive farms, one or more subtypes could be identified by multiplex RT-PCR. Figure 4 gives an overview of the different subtypes detected in the study.

**Figure 5:**
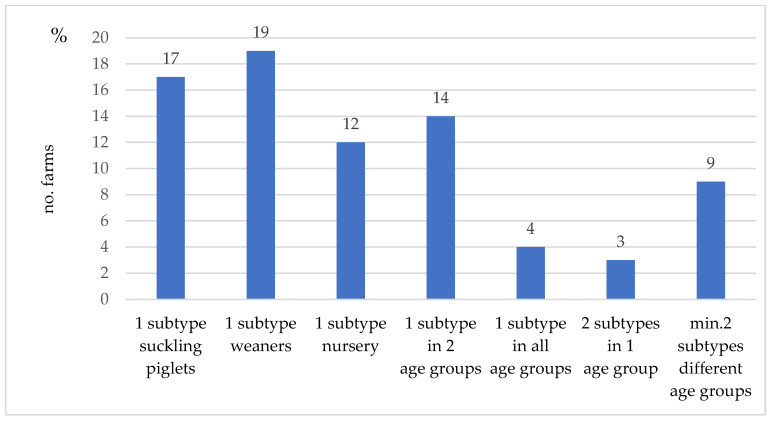
Combinations of IAV subtypes detected/farm/age group in 78 farms, with a minimum of one per multiplex RT-PCR-determined subtype, out of 131 sampled farms.

In 66 of these farms, only one subtype was detected. In 48 farms, this subtype was found in only one of the three investigated age groups; in 14 farms, the subtype was detected in two age groups, and in 4 farms, it was detected in all three age groups. In 12 farms, two subtypes were identified. From these findings, the two subtypes were detected in the same age group in 3 farms, whereas, in the other 9 farms, the subtypes were circulating in different age groups (see the Supplementary Materials for an overview of the results in terms of farms). Furthermore, 2 of these 9 farms were positive for three different subtypes (Figure 5).

### 3.5. IAV Subtyping Results on a Sample Level

Out of the 285 rRT-PCR-positive samples, 165 (57.9%) samples with a Ct value below 30 were tested by multiplex RT-PCR to detect the subtype. The highest subtyping rate was found in the suckling piglets. The majority of all positive samples, 55.8% (48/86), could be subtyped, compared to 54.7% (58/106) and 44.1% (41/93) of subtyped samples in weaners and the nursery, respectively. The different subtypes detected in this study are displayed in Table 3.

## 4. Discussion

Diagnosis of swIAV can be very challenging, particularly in endemically infected herds with rather unspecific clinical signs and a low prevalence within the herd [20]. The use of serologic assays is hampered by the anti-genetic variability of swIAV, leading to cross-reactivity among subtypes. Additionally, antibodies after exposure cannot be discriminated from antibodies formed after vaccination [27,28]. Direct detection by the use of RT-PCR assays is limited by the short course of infection as the virus is mainly shed during days 1 and 5 after infection, and is eliminated from the lungs after 9 days [7,19]. Therefore, the time point of sampling and the selection of the correct pig are crucial for an effective diagnosis. To the knowledge of the authors, this is the first large-scale study investigating sampling approaches in sow farms in a cross-sectional design. To account for variations in infection dynamics due to the different management strategies in the different European countries, farms from 12 European countries were included in our investigations.

The detection rate in sows is regarded as low, due to frequent exposure and the development of immunity [21,29]. Additionally, the duration and the amount of shedding of the virus by sows might be reduced in previously exposed sows. According to Ryt-Hansen et al. (2022), it is more likely to find swIAV-positive suckling piglets than sows in the farrowing unit [30]. Therefore, sows were not included in our sampling protocol.

Until now, the sampling of animals with clinical signs has been propagated. However, in endemic scenarios with rather unspecific clinical signs and a low disease prevalence, finding diseased swIAV-positive animals can be challenging. The results of our study suggest that the approach of focusing on clinically diseased animals must be scrutinized critically as, despite the highest number of animals showing clinical signs being in the nursery, the weaners were significantly more often swIAV-positive, compared to the nursery pigs. The high percentage of nursery pigs with clinical signs may be explained by the fact that swIAV, as part of the PRDC complex [31], predisposes the animals to co-infections. However, as coinfections were not investigated within the scope of our study, the role of coinfections in the evolution of clinical signs in the study farms can only be speculated. Previous research revealed that often, high health-status animals challenged with influenza A viruses alone did not show severe clinical signs, and only mild pathological lesions were detected during necropsy [6,32]. However, coinfections with other pathogens (either bacteria or other viruses) could lead to more severe clinical signs and pathological lesions in the lungs [15,16,18,33]. Thus, in animals showing obvious clinical signs, influenza A virus, which might have been the promoter of the disease, is no longer detectable by PCR due to the fast clearance of the virus.

It has been shown in previous studies that pigs around weaning age are an appropriate target population for swIAV sampling [34,35,36,37]. In accordance with the aforementioned studies, we observed a significantly higher chance of detecting influenza by rRT-PCR in weaners compared to suckling piglets or nursery pigs, which most likely results from the mixing of piglets after weaning, either for transport or within the pens. Furthermore, the additional subsequent spread of the virus in the nursery units, due to airflow or even farm stuff [11], plays a role. The speed of infection depends on the subtypes involved [38], the extent of mixing, and the group sizes within the nursery units [34]. Interestingly, in our study, the weaners were the only group where a closer look at clinical signs increased the chance of finding a positive animal, as diseased animals were significantly more often positive, compared to healthy animals. The occurrence of clinical signs in swIAV-positive weaning pigs can be explained by the decline in MDA at around 5 weeks of age, since the presence of MDA does not prevent infection but does confer clinical protection. Our findings demonstrate, in accordance with previous investigations, the importance of weaners in the infection dynamics of influenza within herds [39,40]. However, sampling strategies should not only focus on weaners but also include suckling piglets as, particularly in endemically infected farms, suckling piglets also play an important role in the maintenance and dissemination of the virus [14,24,41]. Suckling piglets that received maternally derived antibodies (MDA+), either due to the vaccination of the sows or previous infection, will be clinically protected. However, the presence of MDA confers only limited protection against the infection and the spread of the virus, as MDA+ piglets have been shown to shed the virus in the same amounts as animals without MDA (MDA-) [12,13,42]. Therefore, in endemically infected herds, or even on farms where sows are vaccinated and are, thus, providing antibodies to their piglets, the chance of finding influenza A-positive suckling piglets without clinical signs is very high [10,24]. Our data underline this finding, as there was no significant difference in the swIAV-positivity of a farm in terms of vaccination status or the course of disease (endemic/epidemic). It has been shown that the risk of already swIAV-positive suckling piglets is increased by the introduction of unprotected gilts, as they shed vast amounts of the virus when they first get infected shortly before farrowing [30], or due to cross-fostering and the use of nurse sows [43,44]. However, for sampling purposes, it must be considered that suckling piglets may already be infected without exhibiting clinical signs. Our data clearly show that in farms either suspecting an endemic circulation of swIAV or an acute outbreak, the percentage of healthy swIAV-positive suckling piglets (7.2% of all samples) was higher than the percentage of diseased swIAV-positive suckling piglets (4.2% of all samples). As suckling piglets can serve as a reservoir for swIAV, the main aspects that have to be considered to reduce swIAV circulation during the suckling period include the vaccination management of sows and gilts [30,45] and the management of suckling piglets, to minimize transmission [11].

The design of effective control measures for swIAV requires thorough knowledge of all circulating strains on the farm. As the protection of the most commonly used whole-virus-inactivated (WIV) vaccines is primarily linked to the HA [46], therefore, a vaccination program can be implemented, based on HA identification; samples where only the HA could be determined were also included in our analysis. In our study, not all samples with a Ct value < 30 could be successfully subtyped. This may be due to the quality of the specimen or the design of the primers of the subtype determining multiplex RT-PCR. False-negative results often stem from an insufficient match between the primers and the target strain [21,29]. Due to the high genetic diversity of swIAV, the continuous surveillance of new variants is of the utmost importance for the adjustment of PCR assays. The authors intentionally abstained from presenting and analyzing the different subtypes detected in the different countries, due to the low sample sizes of farms in certain countries.

Subtyping was performed to assess the number of subtypes circulating on the farm and to give advice regarding the sampling approach for the detection of all subtypes circulating on the farm. More than one subtype was found in 11 (10.7%) of the positive farms. The simultaneous detection of multiple strains in the same population is not new and has been described before [21,38,39,40,47]. However, we were able to identify different subtypes in different age groups in 9 of our study farms (8.7% of positive farms). Additionally, in most of the farms with a single detection of one subtype, this subtype was only found in one of the three investigated age groups. According to our results, no significant difference in the subtyping rate between the different age groups exists. This strongly indicates that focusing on one age group can lead to a diagnostic gap; the sampling of different age groups is advisable for veterinary practitioners to establish an appropriate control strategy.

The sample size used in this study was calculated using a fixed design prevalence of 15%, for a minimum of 95% probability of detection, and assuming a diagnostic sensitivity and specificity of 90% and 100%, respectively. This led to a target number of 30 sampled animals per farm, which were taken from three age groups [9]. To increase the chance of detecting virus-shedding animals, it was mandatory to sample diseased animals, if available, in the sampled age groups. Based on the high percentage of positive farms (76.8%), it can be assumed that the sample size of 30 animals per farm, allocated to three age groups, was appropriate to detect swIAV in both epidemically and endemically infected farms, under the circumstances of our study. Also farm size did, in contrast to other publications not have an effect on IAV positivity [22,48], allowing the conclusion, that the sample protocol is valid for all farm sizes.

However, it is crucial to consider that all included farms had a history of respiratory distress and/or reproductive failure; thus, in farms with rather nonspecific clinical signs and/or a low intraherd prevalence, a higher sample size is needed to detect the virus. In these cases, the use of group methods can present a cost-effective alternative sampling strategy to testing individual samples. However, in order to identify the subtype/s, and if needed for sequencing purposes, individual samples are superior to group samples [9].

## 5. Conclusions

This article provides new insights into sampling approaches to detect swIAV on sow farms. The results of our investigation indicate that for influenza A diagnosis, the use of 10 nasal swabs each in suckling piglets, weaners, and in the middle of the nursery stage is a valuable tool for influenza detection and the identification of subtypes in endemic and epidemic farms, independent of farm size and the vaccination status of the sows. However, for farms with an expected prevalence of lower than 15%, it is advisable to either increase the individual sample size or use additional group-sampling methods. Furthermore, our study underlines that the sampling of different age groups is mandatory to obtain a comprehensive overview of all circulating variants on a farm. It additionally highlights the fact that sampling strategies should also consider piglets without obvious clinical signs.

## Figures and Tables

**Table 1:** Detection of IAV by RT-PCR at the farm level in the different countries and sampled age groups.

Country	No. of Farms	No. of Positive Farms	Suckling Piglets	Weaners	Nursery
			(1–4 w.o.a.)	(4–6 w.o.a)	(7–9 w.o.a)
			No. of Positive Farms	No. of Positive Farms	No. of Positive Farms
Others *	3	2	2	1	1
Denmark	26	23	12	17	12
France	10	6	2	4	3
Germany	20	17	11	13	10
Ireland	6	5	4	4	3
Italy	19	12	6	6	8
Netherlands	5	4	3	4	3
Poland	7	7	3	6	5
Spain	17	12	8	3	3
UK	18	15	6	10	14
Total	131	103	57	68	62

w.o.a. = weeks of age; * = Belgium, Greece, Hungary.

**Table 2:** Detection of IAV by rRT-PCR on a sample level in the different countries and sampled age group.

	Suckling Piglets	Weaners	Nursery
	(1–4 w.o.a.)	(4–6 w.o.a.)	(7–8 w.o.a)
**Country (No.) Farms**	**no. samples/**	**no. samples/**	**no. samples/**
	**no. positive**	**no. positive**	**no. positive**
Others (*n* = 3) *	5/2	5/1	5/1
Denmark (*n* = 26)	52/17	52/31	51/19
France (*n* = 10)	20/4	19/7	20/3
Germany (*n* = 20)	41/16	36/18	36/16
Ireland (*n* = 6)	12/5	12/7	12/5
Italy (*n* = 19)	42/8	34/6	34/10
Netherlands (*n* = 5)	10/6	10/7	10/5
Poland (*n* = 7)	7/3	8/6	7/5
Spain (*n* = 17)	34/16	34/5	34/4
UK (*n* = 18)	36/9	36/18	36/25
Total: 12 Countries	259/86 ^a,b^	246/106 ^b^	245/93 ^a^

w.o.a. = weeks of age; * = Belgium, Greece and Hungary. Different superscripts between age groups indicate a significant difference (*p* < 0.05) between the number of positive samples.

**Table 3:** IAV Subtypes detected by multiplex RT-PCR in 147 samples with a Ct value < 30 of 749 samples taken from 131 farms.

Subtype	Suckling Piglets	Weaners	Nursery	Total
	(1–4 w.o.a.)	(4–6 w.o.a.)	(7–9 w.o.a.)	
H1avN1	4	8	2	14
H1avN2	11	13	5	29
H1avNx	2	2	1	5
H1huN2	5	4	14	23
H1huN1	4	4	0	8
H1huNx	1	1	2	4
H3N2	2	1	1	4
H3Nx	1	1	2	4
H1pdmN1	9	11	8	28
H1pdmN2	5	2	3	10
H1pdmNx	5	11	3	19
total	49	58	41	148

w.o.a. = weeks of age.

## Data Availability

Data are available from the corresponding author upon request.

## Supplementary Materials

The following supporting information can be downloaded at: https://www.mdpi.com/article/10.3390/vetsci9070338/s1, Table S1: Individual results for each farm, including information about country, course of disease, vaccination status, no. of samples, and no. of positive and subtype viruses detected in each age group of 131 farms tested (w.o.a. = weeks of age).

## References

[1] Er C., Skjerve E., Brun E., Hofmo P.O., Framstad T., Lium B. (2016). Production Impact of Influenza A(H1N1)Pdm09 Virus Infection on Fattening Pigs in Norway1. J. Anim. Sci..

[2] Gumbert S., Froehlich S., Rieger A., Stadler J., Ritzmann M., Zoels S. (2020). Reproductive Performance of Pandemic Influenza A Virus Infected Sow Herds before and after Implementation of a Vaccine against the Influenza A (H1N1)Pdm09 Virus. Porc. Health Manag..

[3] Henritzi D., Petric P.P., Lewis N.S., Graaf A., Pessia A., Starick E., Breithaupt A., Strebelow G., Luttermann C., Parker L.M.K. (2020). Surveillance of European Domestic Pig Populations Identifies an Emerging Reservoir of Potentially Zoonotic Swine Influenza A Viruses. Cell Host Microbe.

[4] Scholtissek C. (1990). Pigs as ‘Mixing Vessels’ for the Creation of New Pandemic Influenza A Viruses. Med. Princ. Pract..

[5] Chastagner A., Hervé S., Quéguiner S., Hirchaud E., Lucas P., Gorin S., Béven V., Barbier N., Deblanc C., Blanchard Y. (2020). Genetic and Antigenic Evolution of European Swine Influenza A Viruses of HA-1C (Avian-Like) and HA-1B (Human-Like) Lineages in France from 2000 to 2018. Viruses.

[6] Everett H.E., Aramouni M., Coward V., Ramsay A., Kelly M., Morgan S., Tchilian E., Canini L., Woolhouse M.E.J., Gilbert S. (2019). Vaccine-Mediated Protection of Pigs against Infection with Pandemic H1N1 2009 Swine Influenza A Virus Requires a Close Antigenic Match between the Vaccine Antigen and Challenge Virus. Vaccine.

[7] Deblanc C., Quéguiner S., Gorin S., Chastagner A., Hervé S., Paboeuf F., Simon G. (2020). Evaluation of the Pathogenicity and the Escape from Vaccine Protection of a New Antigenic Variant Derived from the European Human-Like Reassortant Swine H1N2 Influenza Virus. Viruses.

[8] Detmer S., Gramer M., Goyal S., Torremorell M., Torrison J., Richt J.A., Webby R.J. (2012). Diagnostics and Surveillance for Swine Influenza. Swine Influenza.

[9] Garrido-Mantilla J., Alvarez J., Culhane M., Nirmala J., Cano J.P., Torremorell M. (2019). Comparison of Individual, Group and Environmental Sampling Strategies to Conduct Influenza Surveillance in Pigs. BMC Vet. Res..

[10] Chamba Pardo F.O., Wayne S., Culhane M.R., Perez A., Allerson M., Torremorell M. (2019). Effect of Strain-Specific Maternally-Derived Antibodies on Influenza A Virus Infection Dynamics in Nursery Pigs. PLoS ONE.

[11] Lopez-Moreno G., Davies P., Yang M., Culhane M.R., Corzo C.A., Li C., Rendahl A., Torremorell M. (2022). Evidence of Influenza A Infection and Risk of Transmission between Pigs and Farmworkers. Zoonoses Public Health.

[12] Loeffen W.L.A., Heinen P.P., Bianchi A.T.J., Hunneman W.A., Verheijden J.H.M. (2003). Effect of Maternally Derived Antibodies on the Clinical Signs and Immune Response in Pigs after Primary and Secondary Infection with an Influenza H1N1 Virus. Vet. Immunol. Immunopathol..

[13] Deblanc C., Hervé S., Gorin S., Cador C., Andraud M., Quéguiner S., Barbier N., Paboeuf F., Rose N., Simon G. (2018). Maternally-Derived Antibodies Do Not Inhibit Swine Influenza Virus Replication in Piglets but Decrease Excreted Virus Infectivity and Impair Post-Infectious Immune Responses. Vet. Microbiol..

[14] Ferreira J.B., Grgić H., Friendship R., Wideman G., Nagy É., Poljak Z. (2017). Longitudinal Study of Influenza A Virus Circulation in a Nursery Swine Barn. Vet. Res..

[15] Lin X., Huang C., Shi J., Wang R., Sun X., Liu X., Zhao L., Jin M. (2015). Investigation of Pathogenesis of H1N1 Influenza Virus and Swine Streptococcus Suis Serotype 2 Co-Infection in Pigs by Microarray Analysis. PLoS ONE.

[16] Pomorska-Mól M., Dors A., Kwit K., Czyżewska-Dors E., Pejsak Z. (2017). Coinfection Modulates Inflammatory Responses, Clinical Outcome and Pathogen Load of H1N1 Swine Influenza Virus and Haemophilus Parasuis Infections in Pigs. BMC Vet. Res..

[17] Bougon J., Deblanc C., Renson P., Quéguiner S., Gorin S., Mahé S., Le Dimna M., Barbier N., Paboeuf F., Simon G. (2021). Successive Inoculations of Pigs with Porcine Reproductive and Respiratory Syndrome Virus 1 (PRRSV-1) and Swine H1N2 Influenza Virus Suggest a Mutual Interference between the Two Viral Infections. Viruses.

[18] Saade G., Deblanc C., Bougon J., Marois-Créhan C., Fablet C., Auray G., Belloc C., Leblanc-Maridor M., Gagnon C.A., Zhu J. (2020). Coinfections and Their Molecular Consequences in the Porcine Respiratory Tract. Vet. Res..

[19] Duerrwald R., Schlegel M., Bauer K., Vissiennon T., Wutzler P., Schmidtke M. (2013). Efficacy of Influenza Vaccination and Tamiflu^®^ Treatment—Comparative Studies with Eurasian Swine Influenza Viruses in Pigs. PLoS ONE.

[20] Simon-Grifé M., Martín-Valls G.E., Vilar M.J., Busquets N., Mora-Salvatierra M., Bestebroer T.M., Fouchier R.A., Martín M., Mateu E., Casal J. (2012). Swine Influenza Virus Infection Dynamics in Two Pig Farms; Results of a Longitudinal Assessment. Vet. Res..

[21] Henritzi D., Zhao N., Starick E., Simon G., Krog J.S., Larsen L.E., Reid S.M., Brown I.H., Chiapponi C., Foni E. (2016). Rapid Detection and Subtyping of European Swine Influenza Viruses in Porcine Clinical Samples by Haemagglutinin- and Neuraminidase-specific Tetra- and Triplex Real-time RT—PCR s. Influenza Other Respir. Viruses.

[22] Takemae N., Shobugawa Y., Nguyen P.T., Nguyen T., Nguyen T.N., To T.L., Thai P.D., Nguyen T.D., Nguyen D.T., Nguyen D.K. (2016). Effect of Herd Size on Subclinical Infection of Swine in Vietnam with Influenza A Viruses. BMC Vet. Res..

[23] Martin W. (1997). A Structured Approach for Analysing Survey Data and Making Useful Causal Inferences. Epidémiol. Et St. Anim..

[24] Ryt-Hansen P., Larsen I., Kristensen C.S., Krog J.S., Wacheck S., Larsen L.E. (2019). Longitudinal Field Studies Reveal Early Infection and Persistence of Influenza A Virus in Piglets despite the Presence of Maternally Derived Antibodies. Vet. Res..

[25] Mickey R.M., Greenland S. (1989). The Impact of Confounder Selection Criteria on Effect Estimation. Am. J. Epidemiol..

[26] Hosmer D.W., Lemeshow S. (1989). Applied Logistic Regression.

[27] Reeth K.V., Labarque G., Pensaert M. (2006). Serological Profiles after Consecutive Experimental Infections of Pigs with European H1N1, H3N2, and H1N2 Swine Influenza Viruses. Viral Immunol..

[28] Unterweger C., Debeerst S., Klingler E., Auer A., Redlberger-Fritz M., Stadler J., Pesch S., Lillie-Jaschniski K., Ladinig A. (2021). Herausforderungen bei der Influenzadiagnostik in einem Schweinebetrieb—Ein Fallbericht. Tierärztl. Prax. Ausg. G Großtiere Nutztiere.

[29] Goecke N.B., Krog J.S., Hjulsager C.K., Skovgaard K., Harder T.C., Breum S.Ø., Larsen L.E. (2018). Subtyping of Swine Influenza Viruses Using a High-Throughput Real-Time PCR Platform. Front. Cell. Infect. Microbiol..

[30] Ryt-Hansen P., Nielsen H.G., Sørensen S.S., Larsen I., Kristensen C.S., Larsen L.E. (2022). The Role of Gilts in Transmission Dynamics of Swine Influenza Virus and Impacts of Vaccination Strategies and Quarantine Management. Porc. Health Manag..

[31] Sarli G., D’Annunzio G., Gobbo F., Benazzi C., Ostanello F. (2021). The Role of Pathology in the Diagnosis of Swine Respiratory Disease. Vet. Sci..

[32] Hemmink J.D., Morgan S.B., Aramouni M., Everett H., Salguero F.J., Canini L., Porter E., Chase-Topping M., Beck K., Loughlin R.M. (2016). Distinct Immune Responses and Virus Shedding in Pigs Following Aerosol, Intra-Nasal and Contact Infection with Pandemic Swine Influenza A Virus, A(H1N1)09. Vet. Res..

[33] Pomorska-Mól M., Dors A., Kwit K., Kowalczyk A., Stasiak E., Pejsak Z. (2017). Kinetics of Single and Dual Infection of Pigs with Swine Influenza Virus and Actinobacillus Pleuropneumoniae. Vet. Microbiol..

[34] Chamba Pardo F.O., Schelkopf A., Allerson M., Morrison R., Culhane M., Perez A., Torremorell M. (2018). Breed-to-Wean Farm Factors Associated with Influenza A Virus Infection in Piglets at Weaning. Prev. Vet. Med..

[35] Chamba Pardo F.O., Allerson M., Culhane M., Morrison R., Davies P., Perez A., Torremorell M. (2021). Effect of Influenza A Virus Sow Vaccination on Infection in Pigs at Weaning: A Prospective Longitudinal Study. Transbound. Emerg. Dis..

[36] Diaz A., Marthaler D., Culhane M., Sreevatsan S., Alkhamis M., Torremorell M. (2017). Complete Genome Sequencing of Influenza A Viruses within Swine Farrow-to-Wean Farms Reveals the Emergence, Persistence, and Subsidence of Diverse Viral Genotypes. J. Virol..

[37] Reynolds J.J.H., Torremorell M., Craft M.E. (2014). Mathematical Modeling of Influenza A Virus Dynamics within Swine Farms and the Effects of Vaccination. PLoS ONE.

[38] Rose N., Hervé S., Eveno E., Barbier N., Eono F., Dorenlor V., Andraud M., Camsusou C., Madec F., Simon G. (2013). Dynamics of Influenza A Virus Infections in Permanently Infected Pig Farms: Evidence of Recurrent Infections, Circulation of Several Swine Influenza Viruses and Reassortment Events. Vet. Res..

[39] Nirmala J., Perez A., Culhane M.R., Allerson M.W., Sreevatsan S., Torremorell M. (2021). Genetic Variability of Influenza A Virus in Pigs at Weaning in Midwestern United States Swine Farms. Transbound. Emerg. Dis..

[40] Ferreira J.B., Poljak Z., Friendship R., Nagy É., Wideman G., Grgić H. (2021). Assessment of Exposure to Influenza A Viruses in Pigs between Weaning and Market Age. Vet. Res..

[41] Allerson M., Deen J., Detmer S.E., Gramer M.R., Joo H.S., Romagosa A., Torremorell M. (2013). The Impact of Maternally Derived Immunity on Influenza A Virus Transmission in Neonatal Pig Populations. Vaccine.

[42] Cador C., Hervé S., Andraud M., Gorin S., Paboeuf F., Barbier N., Quéguiner S., Deblanc C., Simon G., Rose N. (2016). Maternally-Derived Antibodies Do Not Prevent Transmission of Swine Influenza A Virus between Pigs. Vet. Res..

[43] Garrido-Mantilla J., Sanhueza J., Alvarez J., Culhane M.R., Davies P., Allerson M.W., Torremorell M. (2021). Impact of Nurse Sows on Influenza A Virus Transmission in Pigs under Field Conditions. Prev. Vet. Med..

[44] Meiners C., Loesken S., Doehring S., Starick E., Pesch S., Maas A., Noe T., Beer M., Harder T., Grosse Beilage E. (2014). Field Study on Swine Influenza Virus (SIV) Infection in Weaner Pigs and Sows. Tierärztl. Prax. Ausg. G Großtiere Nutztiere.

[45] White L.A., Torremorell M., Craft M.E. (2017). Influenza A Virus in Swine Breeding Herds: Combination of Vaccination and Biosecurity Practices Can Reduce Likelihood of Endemic Piglet Reservoir. Prev. Vet. Med..

[46] Grebe K.M., Yewdell J.W., Bennink J.R. (2008). Heterosubtypic Immunity to Influenza A Virus: Where Do We Stand?. Microbes Infect..

[47] Poljak Z., Carman S., McEwen B. (2014). Assessment of Seasonality of Influenza in Swine Using Field Submissions to a Diagnostic Laboratory in Ontario between 2007 and 2012. Influenza Other Respir. Viruses.

[48] Grøntvedt C.A., Er C., Gjerset B., Hauge A.G., Brun E., Jørgensen A., Lium B., Framstad T. (2013). Influenza A(H1N1)Pdm09 Virus Infection in Norwegian Swine Herds 2009/10: The Risk of Human to Swine Transmission. Prev. Vet. Med..

